# Sudden neck swelling with rash as late manifestation of COVID-19: a case report

**DOI:** 10.1186/s12879-021-05911-4

**Published:** 2021-02-27

**Authors:** Caterina Giannitto, Cristiana Bonifacio, Susanna Esposito, Angela Ammirabile, Giuseppe Mercante, Armando De Virgilio, Giuseppe Spriano, Enrico Heffler, Ludovica Lofino, Letterio Salvatore Politi, Luca Balzarini

**Affiliations:** 1 Department of Diagnostic Radiology, Humanitas Clinical and Research Center IRCCS, Via Alessandro Manzoni 56, 20089 Rozzano, Milan, Italy; 2 Paediatric Clinic, Pietro Barilla Children’s Hospital, Department of Medicine and Surgery, University of Parma, 43121 Parma, Italy; 3 Residency Program in Radiology, Humanitas University, Pieve Emanuele, 20072 Milan, Italy; 4 Otorhinolaryngology Unit, Humanitas University, Humanitas Clinical and Research Centre – IRCCS, Rozzano, 20089 Milan, Italy; 5 Department of Biomedical Sciences, Humanitas University, Pieve Emanuele, 20072 Milan, Italy

**Keywords:** COVID-19, Neck swelling, SARS-CoV-2, Case report

## Abstract

**Background:**

Although there are reports of otolaryngological symptoms and manifestations of CoronaVirus Disease 19 (COVID-19), there have been no documented cases of sudden neck swelling with rash in patients with Severe Acute Respiratory Syndrome Coronavirus 2 (SARS-CoV-2) infection described in literature.

**Case presentation:**

We report a case of a sudden neck swelling and rash likely due to late SARS-CoV-2 in a 64-year-old woman. The patient reported COVID-19 symptoms over the previous three weeks. Computed Tomography (CT) revealed a diffuse soft-tissue swelling and edema of subcutaneous tissue, hypodermis, and muscular and deep fascial planes. All the differential diagnoses were ruled out. Both the anamnestic history of the patient’s husband who had died of COVID-19 with and the collateral findings of pneumonia and esophageal wall edema suggested the association with COVID-19. This was confirmed by nasopharyngeal swab polymerase chain reaction. The patient was treated with lopinavir/ritonavir, hydroxychloroquine and piperacillin/tazobactam for 7 days. The neck swelling resolved in less than 24 h, while the erythema was still present up to two days later. The patient was discharged after seven days in good clinical condition and with a negative swab.

**Conclusion:**

Sudden neck swelling with rash may be a coincidental presentation, but, in the pandemic context, it is most likely a direct or indirect complication of COVID-19.

## Background

Non-traumatic sudden neck swelling is an emergency condition resulting from infectious and non-infectious conditions, and the emergency physicians and otolaryngologists should be able to determine its etiology. The most common causes include acute infectious and inflammatory diseases [1]. The differential diagnoses are acute sialadenitis, bacterial or viral cervical lymphadenitis, cellulitis, infected cyst, and neck hemorrhage from carotid blowout syndrome or extraglandular bleeding due to an underlying thyroid or parathyroid disease [2].

To date otolaryngologists have observed an increase in the number of patients with acute parotitis [3, 4] and submandibular gland sialadenitis [5], which could be related to CoronaVIrus Disease 19 (COVID-19) when associated to reported general and otolaryngologic symptoms, including fever, anorexia, arthralgia, myalgia, headache, fatigue, nasal obstruction, rhinorrhea, postnasal drip, sore throat, face pain, and loss of smell and taste [6]. Similarly, retropharyngeal fluid collection [7] and peritonsillar or parapharyngeal abscess [8, 9] have been reported among the unique presentations of COVID-19.

To our knowledge, COVID-19 presenting with primary unilateral sudden neck swelling and rash due to edema of subcutaneous tissue, hypodermis, and muscular and deep fascial planes changes is unreported in the literature. We report our case highlighting this unusual presentation occurring in the context of severe acute respiratory syndrome coronavirus 2 (SARS-CoV-2) infection.

## Case presentation

A 64-year-old woman arrived at our emergency department with a sudden left lateral cervical swelling associated with dyspnea and transient paresthesia of the left face with speech difficulties. The patient ruled out any exposure to food allergies, drugs, trauma o insect sting, and presented symptoms suggestive of COVID-19: axillary temperature of 37.5°, dysgeusia, and cough over the previous three weeks partially resolved in 7 days. Her husband had died of COVID-19 three weeks before. Her past medical history included arterial hypertension and surgically-treated esophageal achalasia without any history of allergies.

**Fig. 1:**
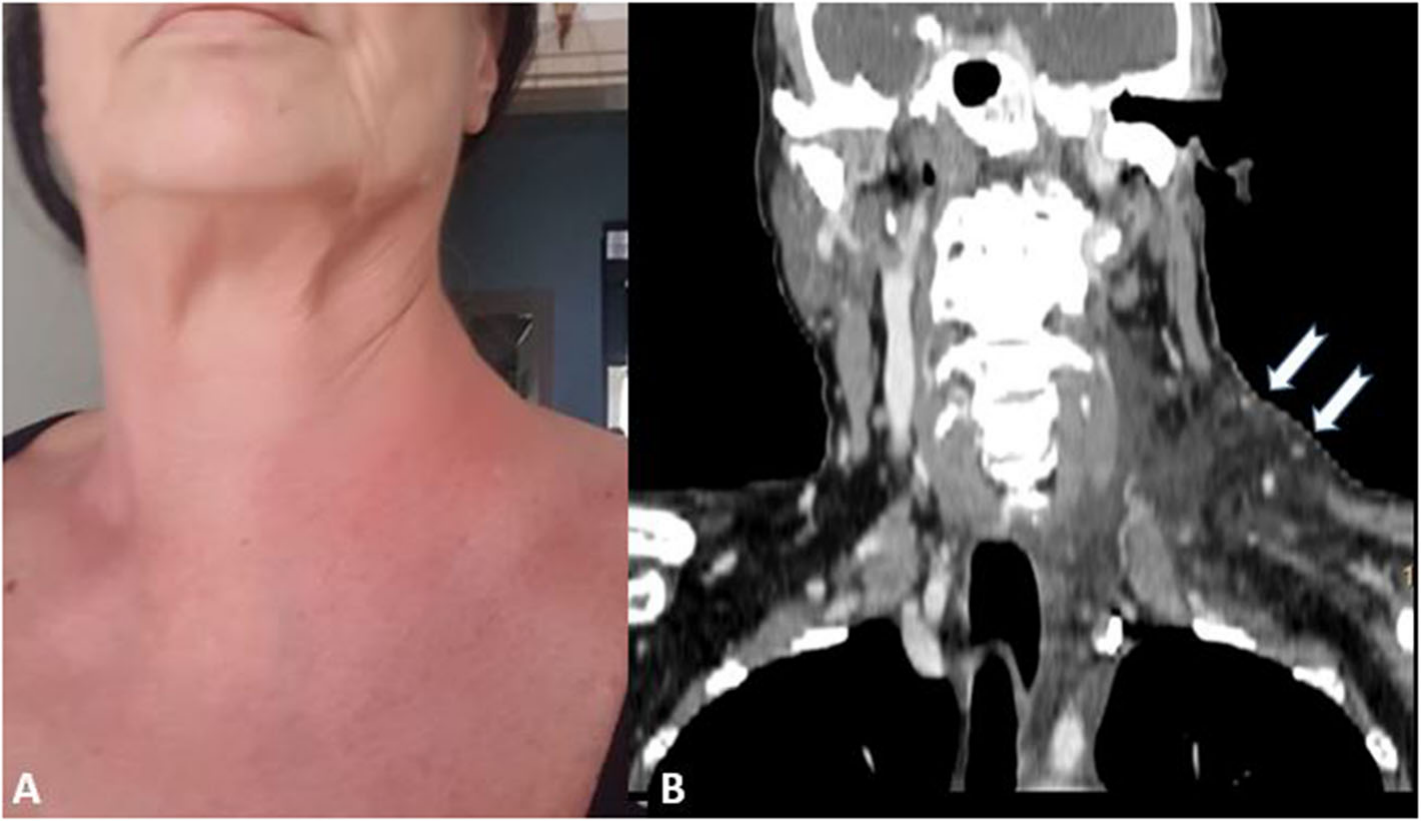
A 64-year-old woman with a sudden left lateral cervical swelling (A) and the corresponding coronal multiplanar reconstruction (MPR) contrast-enhanced CT images at the admission (B) showing the soft-tissue swelling (white arrows)

The physical examination revealed a left mobile edematous swelling in the neck. The skin of the neck was reddish and warm (Fig. 1a). Oxygen saturation was 98%, temperature measured by a tympanic thermometer was 36°, chest and neurological examinations were normal.

Laboratory tests showed C reactive protein of 0.43 mg/dL (normal value < 0.50 mg/dL), white blood cells count of 11, 39 × 10^3/mm^3, neutrophils 71%, eosinophils 2%, D-Dimer 750 ng/mL (normal value 200–350 mg/dL); the remaining values were unremarkable.

**Fig. 2:**
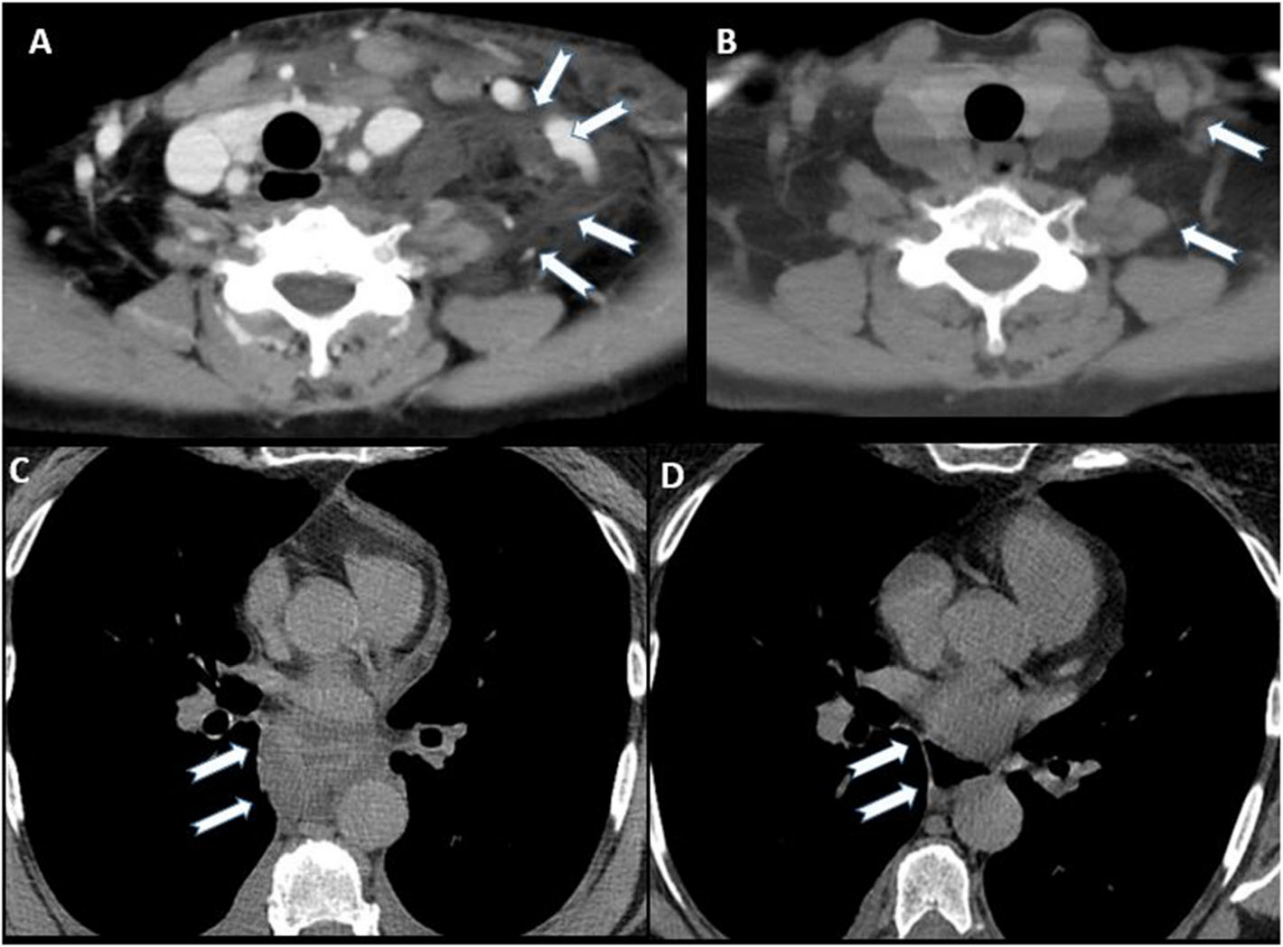
Computed tomography images. Axial contrast-enhanced and non-contrast neck CT images on the admission and discharge (A,B) showed soft-tissue swelling and diffuse reticulation of subcutaneous fat with edema around vessels (arrows in A) with a complete resolution in few days (arrows in B). Axial contrast-enhanced and non-contrast chest Computed Tomography images on the admission and discharge (C,D) documented esophageal wall edema with adjacent effusion (arrows in C) with a complete resolution in few days (arrows in D)

Neck Computed Tomography (CT) revealed a diffuse soft-tissue swelling and edema of subcutaneous tissue, hypodermis, and muscular and deep fascial planes (Fig. 1 and 2b and a) with normal appearance of nodes and salivary glands. The chest scans also revealed bilateral subpleural ground-glass opacities and lung pleural effusion. Collaterally, CT documented esophageal wall edema with adjacent effusion (Fig. 2c). A nasopharyngeal swab was performed and yielded a positive result for SARS-CoV-2 by Polymerase Chain Reaction (PCR).

She was immediately hospitalized and started therapy with lopinavir/ritonavir (200 mg and mg respectively) twice a day and hydroxychloroquine (200 mg) twice a day, combined with piperacillin/tazobactam (4000 mg and 500 mg,respectively) by intravenous administration three times a day for 7 days as empiric therapy of deep neck infection. The neck swelling resolved in less than 24 h, while the erythema was still present up to two days later. Chest and neck CT performed four days later confirmed resolution of the swelling and esophageal edema (Fig. 2b and d). The patient was discharged after seven days in good clinical condition, and PCR test was negative for SARS-CoV-2 on the twenty-first day.

## Discussion and conclusions

This case shows that SARS-CoV-2 infection could present with a sudden neck swelling associated with pneumonia and rash, highlighting the need of an appropriate differential diagnosis in sudden lateral swelling of the neck in a pandemic context..

In fact, a sudden lateral swelling of the neck can be caused by an acute inflammatory reaction due to various pathologies such as lateral neck cyst infection, neck abscess, or retropharyngeal abscess, soft-tissue edema, aneurysm, submandibular gland swelling, and thyroid or parathyroid gland diseases and laryngocele [3]. In our case, CT revealed a diffuse soft-tissue swelling and diffuse fat changes with edema of subcutaneous tissue, hypodermis, and muscular and deep fascial planes. These findings are not specific and can be seen in other entities such as inflammatory fasciitis, cellulitis, post-radiation changes, ruptured cysts, inflammatory myositis, trauma, lymphedema, and vasculitis [3]. Lymph-nodes and salivary glands were not enlarged. In consideration of the clinical presentation of rubor (redness), tumor (swelling), calor (heat), and the imaging findings, cellulitis (CFC), inflammatory myositis, and vasculitis were first considered in the differential diagnosis. Oro-dental and oropharyngeal causes of infection were also excluded. Both the anamnestic history of the patient’s husband who had died of COVID-19 and the collateral findings of pneumonia and esophageal wall edema suggested the possible association with COVID-19 disease. This was confirmed by nasopharyngeal swab PCR. Unfortunately, the rapid resolution of the swelling did not allow biological sampling for virus isolation and citology characterization.

It is difficult to verify in which context cellulitis, inflammatory myositis, or vasculitis could occur in terms of SARS-CoV-2 infection. Various theories have been developed: circulating viral particles in the cutaneous blood vessels could be responsible for a lymphocytic vasculitis or the immune response to viral infection could activate Langerhans cells, causing vasodilation and spongiosis [10]. The angiotensin-converting enzyme 2, receptor for SARS-CoV-2 is present on endothelial cells in multiple organs, including dermal blood vessels and arterial smooth muscle cells [10]. Despite the absence of a definitive causal relationship, this interaction could explain COVID-19-endothelitis with systemic impaired microcirculatory function in different vascular beds which could be the cause of the neck swelling and esophageal wall edema reported in our case .

Most Sars-CoV-2 infected patients arrived at respiratory clinic or emergency clinic. A patient arriving at an emergency department with an unusual symptoms, such as the presentation of a sudden neck swelling, may not receive the prompt diagnosis and therapy they required, thereby also increasing the medical staff’s exposure to Sars-Cov-2.

Our case shows the possibility of considering SARS-CoV-2 infection in the differential diagnosis of sudden neck swelling, taking into consideration other COVID-19 typical history and symptoms. This presentation may be coincidental, but, in the described context, it is most likely a direct or indirect complication of COVID-19. Sudden neck swelling can be added to the growing list of potential complications of COVID-19 and it will be interesting to see if other similar cases emerge over time. Recently, Gianotti et al. [11] supposed that a woman presented with urticarial plaque-like dermatosis on the arms and a mild sore throat could be the dermatological Italian patient zero as confirmed by immunohistochemical investigations for SARS-CoV-2 nucleocapsid antigens on paraffin sections.

Although further studies on the association between neck swelling and SARS-CoV-2 are needed, our observation could help emergency physicians to avoid misdiagnosis by considering sudden neck swelling with a rash as a possible sign of COVID-19 when all the other differential diagnoses have been ruled out in a pandemic context.

## Data Availability

*The datasets used and analysed during the current study is available from the corresponding author on reasonable request.*

## Abbreviations

COVID-19: CoronaVirus Disease 19
SARS-CoV-2: Severe Acute Respiratory Syndrome Coronavirus 2
CT: Computed Tomography
PCR: Polymerase Chain Reaction

## References

[1] Giannitto C, Esposito AA, Casiraghi E, Biondetti PR (2014). Epidemiological profile of non-traumatic emergencies of the neck in CT imaging: our experience. Radiol Med.

[2] Kamalian S, Avery L, Lev MH, Schaefer PW, Curtin HD, Kamalian S (2019). Nontraumatic head and neck emergencies. Radiographics..

[3] Lechien JR, Chetrit A, Chekkoury-Idrissi Y, et al. Parotitis-Like Symptoms Associated with COVID-19, France, March–April 2020. Emerg Infect Dis. 2020 26(9): 10.3201/eid2609.202059. doi: 10.3201/eid2609.202059 10.3201/eid2609.202059 PMC7454100 32491984

[4] Fisher J, Monette DL, Patel KR, Kelley BP, Kennedy M. COVID-19 associated parotitis. Am J Emerg Med. 2021; 39: 254.e1–254.e3. doi: 10.1016/j.ajem.2020.06.059. 10.1016/j.ajem.2020.06.059 PMC7320680 32631770

[5] Chern A, Famuyide AO, Moonis G, Lalwani AK (2020). Sialadenitis: a possible early manifestation of COVID-19. Laryngoscope..

[6] Mercante G, Ferreli F, De Virgilio A, et al. Prevalence of Taste and Smell Dysfunction in Coronavirus Disease 2019 [published online ahead of print, 2020 Jun 18]. JAMA Otolaryngol Head Neck Surg. 2020; e201155. doi: 10.1001 /jamaoto.2020.1155. 10.1001/jamaoto.2020.1155 PMC7303892 32556070

[7] Steehler AJ, Ballestas SA, Scarola D, Henriquez OA, Moore CE (2020). Observation of retropharyngeal fluid collection in 2 COVID-19 positive patients. Ear Nose Throat J.

[8] Sideris AW, Ghosh N, Lam ME, Mackay SG (2020). Peritonsillar abscess and concomitant COVID-19 in a 21-year-old male. BMJ Case Rep.

[9] Ajeigbe T, Ria B, Wates E, Mattine S. Severe parapharyngeal abscess that developed significant complications: management during the COVID-19 pandemic. BMJ Case Rep. 2020 22;13(12):e236449. doi: 10.1136/bcr-2020-236449. 10.1136/bcr-2020-236449 PMC10577725 33370968

[10] Novak N, Peng WM, Naegeli MC, et al. SARS-CoV-2, COVID-19, skin and immunology - what do we know so far? Allergy. 2020. 10.1111/all.14498. 10.1111/all.14498 PMC7404682 32658359

[11] Gianotti R, Barberis M, Fellegara G, et al. COVID-19 related dermatosis in November 2019. Could this case be Italy's patient zero? Br J Dermatol 2021; 10.1111/bjd.19804. 10.1111/bjd.19804 PMC9619443 33410129

